# WELL-BEING BEFORE AND DURING THE COVID-19 PANDEMIC: ASSOCIATIONS WITH RESILIENCE AND CHRONIC CONDITIONS

**DOI:** 10.1093/geroni/igac059.2394

**Published:** 2022-12-20

**Authors:** Jay Kayser, Jacqui Smith

**Affiliations:** University of Michigan, Ann Arbor, Michigan, United States; University of Michigan, Ann Arbor, Michigan, United States

## Abstract

Adults over the age of 60 tend to have higher well-being than younger adults, and high well-being is associated with a range of favorable mental and physical health outcomes. Resilience is related to the maintenance of well-being in the presence of challenging circumstances and stressors. Chronic health conditions are common in older adults and represent a stressor. Using longitudinal data, we address two research questions: 1) Are chronic health conditions and resilience predictive of changes in well-being before and during Covid-19? 2) Does the number of chronic health conditions and resilience interact in predicting changes in well-being before and during Covid-19? To answer these questions, we analyzed data from participants in the Health and Retirement Study (HRS) who completed both the 2016 and 2020 waves (N = 2276). On average, these participants were age 71.44 (SD = 7.35) and reported 2.40 chronic conditions. In unadjusted models, both the number of chronic conditions (β = -.21) and resilience (β = .09) were related to changes in well-being (R2 = .32). When covariates were added, these values were attenuated but remained statistically significant. The interaction between resilience and chronic health conditions was not statistically significant. Though the number of chronic health conditions and resilience play an important role in well-being for older adults, high resilience does not moderate the relationship between the number of chronic health conditions and changes in well-being. Further investigations using longitudinal data are needed to understand the relationship between resilience and well-being for those with chronic health conditions.

